# Taking Stock: An Adaptable Research and Partnership Model for Developing Puberty Education in 10 Countries

**DOI:** 10.9745/GHSP-D-22-00498

**Published:** 2023-06-21

**Authors:** Marni Sommer, Margaret L. Schmitt

**Affiliations:** a Mailman School of Public Health, Columbia University, New York, NY, USA.

## Abstract

There is a growing global interest in puberty and early adolescence and the importance of ensuring young people have the information and support they need during this critical phase of transition in the life course. This article highlights an adaptable model being used to support the development of contextually appropriate puberty education, in the form of illustrated and often bilingual books, for early adolescent girls and boys in countries around the world. This youth-centered participatory research and design model, which relies on the generation of community and government partnerships, has been employed in 10 countries thus far. Valuable learning has been documented from various contexts, including the approach’s flexibility in adapting to the inputs of government and community members, incorporating local buy-in as a key ingredient for sustainability, using in-country experts for social and cultural appropriateness of illustrations and translations, and ensuring that the authentic youth voices captured through the participatory data collection and field-testing shape the educational content. The continuous integration of insights and perceptions of adults who influence young people’s lives into the development process is also essential to enabling the uptake of puberty content in each new country. Parents, educators, youth service providers, and government officials are often the gatekeepers to young adolescents receiving puberty content and are thus critical to the process. This review of more than a decade of experience using this model underscores the essentiality of 2 key components—local partnership and participatory data collection—and highlights the importance of flexible approaches that are adapted to the unique sociocultural and environmental conditions in each country context.

## Abstract (precis)

Puberty books, developed through participatory youth engagement and local partnerships, could provide a meaningful and culturally acceptable tool to inform youth of puberty content in areas where educational systems cannot do so.

## Teaser

**Key Findings:**

- Puberty books serve a valuable role in reaching youth in areas where educational systems do not deliver such content.
- It is essential to keep youth at the center of the content development process to produce meaningful and culturally acceptable books.
- More effort is needed to expand puberty content globally to ensure parents, educators, and youth-focused practitioners understand the value of puberty education and feel capacitated to convey it.

**Key Implications:**

- Program managers should consider using existing puberty books or developing new books in countries where they work to educate youth and build their confidence in their changing bodies.
- Policymakers should consider incorporating locally developed puberty books as government-approved supplementary readers into their educational systems to support healthy adolescent transitions, particularly in contexts that lack routine delivery of puberty content in schools.
- Educators should consider sharing puberty books with students in late primary and early secondary school, and possibly parents and caregivers, to facilitate learning and communication, reduce fear and shame on what can be sensitive topics, and encourage interest in reading.

## INTRODUCTION

There is a growing global focus on puberty and early adolescence (aged 10–14 years) and the importance of assuring that young people receive education and support during this critical life phase in which they experience rapid physical, psychosocial, and cognitive developments.^1^^,^^2^ For girls, this phase includes adjusting to a range of social and physical developmental changes, which may include a change in their role in the family and a reduction in their freedom of movement, as well as the onset of menstruation, which studies around the world have identified as a significant life event for which many girls are inadequately prepared.^3^^–^^5^ For boys, about whom there is much less documented, the onset of puberty can bring similarly profound social and physical changes, such as wet dreams (semenarche) and peer pressures to engage in risky behaviors but with even more silence imposed on their need for guidance.^6^^,^^7^ For all young people, a healthy transition through adolescence helps to reduce confusion and uncertainty and normalize the changes of puberty, along with setting the course for longer-term positive health outcomes, including reduced vulnerability to early pregnancy, sexual or physical violence, and the uptake of drugs and alcohol.^8^^,^^9^ Although the sociocultural context surrounding the transition into young adulthood can vary significantly in different parts of the world, puberty is a universally experienced phase of significant emotional and physiological transition, and it requires intervention and support, such as information on bodily changes and reassurance of the normalcy of those changes.^10^^,^^11^ The Global Early Adolescence Study conducted in 11 countries further highlights the importance of addressing gendered norms in early adolescence,^12^ the stage of life where gender norms are shaped, thus, emphasizing the need to deliver both behavioral and attitudinal interventions.^13^

Puberty is a universally experienced phase of significant emotional and physiological transition that requires intervention and support.

Nevertheless, there continues to be insufficient attention in the form of research, resources, and prioritization globally to providing early adolescents with appropriately timed guidance and information about what to expect and how to adjust to the many social, emotional, psychological, cognitive, and physical changes of puberty.^1^^,^^2^^,^^14^ One explanation is that the transition into young adulthood, with its accompanying social and cultural expectations, has traditionally been perceived as belonging to the private sphere or family.^15^^,^^16^ However, growing recognition of the inadequacy of this approach suggests that the global health and education communities are overdue to address the issue of puberty more systematically and comprehensively, starting with the education of young people around the world. Although sexual health education and other curricula in schools exist in many countries, such efforts often fall short of including puberty content beyond the biology of human development or the practicalities of adjusting to this important life phase that establishes a foundation for a healthy future. This may include, for example, girls understanding how to manage their menstrual periods or boys feeling it is acceptable to ask questions about their mood fluctuations. Furthermore, puberty education programs globally are rarely rigorously evaluated, particularly if embedded within broader sexual health curricula.^1^ The incorporation of puberty education into sexual health curricula, including comprehensive sexuality education, may also hinder the appropriate timing of its delivery, given it is often included in life skills or other programming aimed at older adolescents (aged 15–19 years). In addition, such content delivery may not take into account the evidence of younger age of pubertal onset,^17^ the need for education in primary school, and parent or education sector hesitation to cover sexual health topics perceived to be sensitive within local society.

Education is a powerful tool. Although in and of itself, puberty education may not be sufficient, it is essential—and a basic human right—to ensure that young people understand what is happening within their bodies, including their changing emotions and relationships, and the sociocultural reactions of those around them as their bodies mature into those of young adults.^18^ It is also critical for normalizing the behavior of asking for help during what is a confusing time for many, including enabling young people to feel empowered to reach out for support as needed or in solidarity with peers when adjusting to the new body changes or social reactions experienced.^19^ One approach to providing puberty education over the last decade has been an adaptable model for puberty book development conducted in partnership with ministries of education, ministries of health, and other key stakeholders in 10 countries. The overarching aims of the book include youth development, health and well-being, literacy, and self-efficacy. Central to this effort is the engagement of young people in the process and an investment in the local economies of each country through the hiring of local illustrators, translators, and publishing companies. After 10 years of developing puberty books, with over 2 million copies distributed (not including digital versions), it is important to take stock and reflect on lessons learned.

## PUBERTY BOOK DEVELOPMENT PROCESS

The model aims to guide the development of a puberty book (or books) that informs young people about their changing bodies and provides practical knowledge and guidance on how to adapt to puberty and manage new experiences, such as menarche and wet dreams. Additionally, the book seeks to help youth feel more confident and capable to ask for support if needed from teachers, family members, or health care workers; engage with their peers about these changes; and communicate more openly with adults in their lives, such as parents and caregivers. A central tenet of each book is to provide context-specific guidance and experiences directly from older youth to younger youth who have yet to reach puberty, with older adolescents’ advice captured in personal narratives through data collection that are ultimately published within each book. The book also aims to encourage a love of reading, with the hope that the content, of interest to most young people around the world, will contribute to fostering an enjoyment of learning from books.

The model aims to guide the development of a book that informs young people about their changing bodies and provides guidance on how to adapt and manage new experiences.

In each country, a 5-step systematic model (Table 1) is used to develop a new puberty book or books. The steps include: (1) engage local key stakeholders and obtain formal approvals; (2) conduct participatory research with adolescent girls or boys; (3) draft the written book content for key stakeholder inputs and have a local illustrator, translator, and publisher develop an illustrated and translated draft; (4) field-test with youth, parents, teachers, and stakeholders; and (5) distribute the book and submit for review. Pilot copies are subsequently distributed (10,000–15,000 copies) to capture initial feedback and learning while the book is submitted for review by the local government for approval as a supplementary reader in primary and early secondary schools.

**TABLE 1.** The 5 Steps of the Puberty Book Development Model

**Step**	**Description of Activities and Objectives**
1. Engage stakeholders and obtain formal approvals	**Objective**: To generate buy-in from the government and identify appropriate data collection sites, usually 1 urban and 1 rural site in each country. **Activities**: Examine existing puberty resources in the select country, including meeting with key government stakeholders (e.g., ministry of education, ministry of health) to determine if a gap in puberty education exists and that a book would serve as a useful contribution. Capture inputs from additional actors, such as youth-serving NGOs, local researchers, and advocates knowledgeable about youth issues.
2. Conduct participatory research	**Objectives**: To capture adolescent stories about growing up, questions about puberty, and recommendations for what other girls and boys need to know as they reach puberty; to capture adults’ perspectives on beliefs and issues of importance. **Activities**: Conduct participatory activities with adolescent girls and/or boys (aged 15–19 years) in and out of school. Interview adults (e.g., parents, teachers, health workers, and religious leaders) who interact in young peoples’ lives in the local context.
3. Draft book content	**Objective**: To include content that combines basic puberty guidance grounded in local education curricula, a selection of written stories directly authored by girls and boys, and a series of questions and facts. **Activities**: Draft written content. Local stakeholders review draft and provide inputs. A local illustrator, translator, and publisher develop an illustrated draft of the book for field-testing.Note: In many countries, the books are dual language, with the languages being taught in schools on the same page to enable improved comprehension. The language selection for the book is carefully determined by the local government. Hiring of local talent supports investment in the local economy and ensures that the content is socially and culturally appropriate to the context.
4. Field-test and finalize	**Objectives**: To ensure the book content is appropriate and meaningful to youth and acceptable to the adults in their lives, including parents, government, and key stakeholders; and to ensure the literacy level is correct for the average reader and that youth feel that the images align with the words on each page. **Activities**: Field-test with girls or boys aged 10–14 years; significant time is spent reviewing each page for the illustration and text used. Youth-recommended edits are prioritized when finalizing the book content in each context. Field-test with primary school teachers, parents or caregivers, health and education members of government, and other relevant stakeholders.
5. Distribute and review	**Objective**: To disseminate the book. **Activities**: In many countries, 10,000–15,000 pilot book copies were distributed to local stakeholders that supported the book development process, along with other youth actors (e.g., schools and youth centers that participated in the data collection, youth-serving NGOs, UN agencies, and government champions). Submit the book for review by government education bodies as a supplementary reader in schools. In some countries, after approval has been received, additional translations of the book(s) are created, usually by government request.Note: In terms of their usage, the books are designed for young people to receive and read on their own, ideally taking them home to share with siblings, parents, or family members or youth who are out of school. However, in some contexts, NGOs or schools have opted to incorporate the books into clubs or programming, with teachers or peer educators interacting with youth as they read.

Abbreviations: NGO, nongovernmental organization; UN, United Nations.

An adaptation process underpins the approach in each new country, thus ensuring the book maintains the core puberty content of each country’s curricula along with customized content (Table 2). This content includes, for example, context-specific first menstrual period or peer pressure stories directly captured from youth, puberty questions and local beliefs or myths, and locally developed illustrations. Books are never simply translated for a new context, as the adaptation process and generation of country-level buy-in are critical for uptake, scaling, and ultimate success (Box).

**TABLE 2.** Illustrative List of Participatory Activities Used With Girls and Boys

**Activity Type**	**Description**	**Adaptation for Girls**	**Adaptation for** **Boys**
Individual (boys and girls)	**Puberty questions**: Girls and boys are asked to write 3 anonymous questions they have about puberty or body changes. The team provides responses to the questions during subsequent group sessions.		
	**Story writing**: During different sessions, each youth is given prompts for writing anonymous stories about puberty experiences, including their advice for younger youth. **Note**: Some of the story writing requires preparatory activities, such as explaining the meaning of “peer pressure.”	In most countries, girls write 1 story. **Menstrual stories**: Girls are asked to write a 1-page story about their first period, including how they felt, how they managed, who they told, and advice for younger girls. **Note**: One country book included peer pressure stories for girls.	In most countries, boys write 3 stories. **Body change stories**: Boys are asked to write a 1-page story about erections or wet dreams, including how they felt when they experienced it for the first time, how they managed, who they told, and advice for younger boys. **Note**: Boys also write stories about experiences of peer pressure and of engaging in or witnessing violence.
	**Myths about puberty**: Each girl or boy is asked to write down the period or puberty myths that they have heard.	Instructions focus on myths about menstruation.	Instructions focus on myths about all types of puberty body changes.
Group (girls only)	**Drawing**: In small breakout groups, girls are asked to draw and label what a “girl friendly” toilet at school would appear like.		
	**100 million [insert local currency]**: In small breakout groups, girls imagine that they have an enormous amount of local currency and use it to list all the ways they would improve the school environment for girls experiencing puberty and menstruation.

BOX Example Base Outline for a Puberty Book^a^

1. Definitions, concepts, and explanations of puberty signs and experiences, including attention to physical, emotional, and social changes.
2. Stories written by adolescents in each country covering a range of topics, such as first menstrual periods and experiences with peer pressure and gender-based violence.
3. A review of answers to commonly asked questions anonymously submitted by adolescents in each country.
4. Puberty guidance on how to manage pubertal changes that aligns with cultural norms and practices of that country (e.g., how to manage your menstruation, new body odors, and hair growth).
5. Annotated body changes diagrams.
6. A review of true and false questions and local myths and beliefs surrounding puberty.

^a^Complete book PDF files are available for free at www.growandknow.org.

An important parallel aspect of the puberty book model in some contexts has been to contribute to building the evidence on early adolescence transitions and young adulthood, including new risks and vulnerabilities that arise for youth along with their own voiced recommendations for how they could have been better informed and supported in advance of the changes of early adolescence (Box). This has required partnering with local research institutions, acquiring ethical approvals, and analyzing and writing up the findings for peer-reviewed publications (Table 3).^6^^,^^7^^,^^14^^,^^16^^,^^20^^–^^31^ Scientific publishing ensures that the range of insights directly gathered from youth about growing up in different countries are shared, beyond what may be included in the puberty book. It is also important to note, as exemplified by the Blake et al. article that describes an evaluation conducted after Ethiopian school girls read the puberty book, how simply reading the book increased their knowledge; decreased their fear, confusion, and shame; and encouraged them to seek out the adults in their lives with questions.^21^ A quasi-experimental design of the U.S. girl’s puberty book is currently underway, using a similar approach examining how a very low capacity needs approach—giving the girls the book to read on their own—may or may not impact their knowledge, attitudes, and confidence about puberty.

**TABLE 3.** Illustrative List of Peer-Reviewed Puberty Evidence Generated From the Model

**Country**	**Peer-Reviewed Evidence**
Cambodia	Scandurra et al., 2016^6^ Connolly and Sommer, 2013^20^
Ethiopia	Blake et al., 2017^21^ Smiles et al., 2017^22^
Ghana	Sommer and Ackatia-Armah, 2012^23^
Madagascar	Sommer et al., 2020^24^
Pakistan	Mumtaz et al., 2019^25^
Tanzania	Sommer et al., 2015^7^^,^^a^ Sommer et al., 2014^14^^,^^a^ Teizazu et al., 2023^26^ Sommer, 2011^27^ Sommer, 2009^28^^,^^a^
USA	Schmitt et al., 2022^29^ Schmitt et al., 2022^30^ Schmitt et al., 2021^31^
Cross-country	Sommer et al., 2015^16^

a The studies leading to these articles included the integration of the methodologies aimed at the book development in each country but were also part of larger studies with additional funding sources.

To date, after the final phase or Step 5 (Table 1) was completed, almost all of the country books were approved by the relevant government authority, and United Nations (UN) agencies, governments, and nongovernmental organizations (NGOs) subsequently began to order copies. As of early 2023, more than 2 million books have been distributed globally. In 2012, a partnership was also established with Worldreader, a global NGO focused on providing free access to digital books in low-resource contexts.^32^

## COUNTRY SNAPSHOTS

Adapting the methodology to each country context is a critical aspect of the puberty book model approach. Although the 5 steps remain consistent across contexts, there have been differences in local partners and in the country-specific and global issues that have shaped data collection, book development efforts, and distribution. The latter, in particular, is left to the discretion of the NGOs, school systems, and UN agencies that distribute the books, including the evaluation of impact. We describe some country examples that demonstrate adaptation within the 5 steps of the model and subsequent local uptake of the book(s).

Adapting the methodology to each country context is a critical aspect of the puberty book model approach.

### Tanzania Girl’s and Boy’s Puberty Books

As the country for the first girl’s puberty book (2009) and boy’s puberty book (2012), Tanzania provided useful insights for the methodologies developed to capture learning from youth and from the local partnerships that supported the process. Both books were developed in partnership with government colleagues at the Tanzania Ministry of Education and Vocational Training, along with those at the local offices of UNICEF, United Nations Population Fund, and NGOs. The boy’s book, completed after the girl’s book, was produced in partnership with Muhimbili University of Health and Allied Sciences (Tanzania). Having local health expert coauthors was crucial given the novelty of creating content for boys. Over half a million books have been distributed across Tanzania, demonstrating how sustained advocacy and informal networks influence uptake of usage of the books by primarily NGOs working in various parts of the country, along with government and UN agency distribution. The puberty books were also translated into Braille by local organizations for distribution to visually impaired students. In 2019, a new partnership with a local animation studio, TAI Tanzania, led to the development of a promotional video to support book dissemination efforts. One key challenge has been changing conditions in the local publishing industry, resulting in the book now with its fourth publishing company in 12 years.

### Cambodia Girl’s and Boy’s Puberty Books

The Cambodian books provide a useful example of strong government buy-in. Upon completion of the girl’s book, the Cambodian government asked UNICEF and United Nations Population Fund to support the distribution of copies to school libraries across the country and for Grow & Know to create a boy’s puberty book. The Cambodia boy’s book was subsequently developed in partnership with the Kampuchean Action for Primary Education, an NGO that had been involved in the girl’s book development process. Over the past decade, the government has annually requested UN support to continue the distribution of additional books to school libraries countrywide.

### Madagascar Girl’s Puberty Book

Madagascar was the first country book project initiated by a local youth-focused NGO (Projet Jeune Leader), whose leadership reached out about collaborating on a new book. This proved a useful adaptation of the model, with Projet Jeune Leader obtaining government permissions and leading data collection. Grow & Know virtually trained the research team and provided book development guidance. This was the first book provided in a single language, Malagasy, at the request of the local partner and the government. New content was also added to the girl’s book, including information on how to use menstrual products and a diagram of the female reproductive system. Project Jeune Leader subsequently incorporated usage of the puberty book into their sexual and reproductive health school-focused programming.

### Pakistan Girl’s Puberty Book

The Pakistan girl’s book was conducted in partnership with a local NGO, the Real Medicine Foundation, and the University of Alberta School of Public Health (Canada). The fieldwork proved to be complex given the more conservative nature of the teachers encountered, who were initially resistant to discussing puberty, and due to security issues that limited entering schools for data collection. Given the decentralized nature of the Pakistan education system, the team was advised to apply for regional government approval for each province rather than national approval—the first country in which this approach was taken. In seeking approval for the book’s overall content, Pakistan provided a useful example of where more conservative beliefs, including not mentioning pregnancy, were part of a compromise approach to using the books in school with early adolescents.

### Ethiopia Girl’s Puberty Book

In Ethiopia, a girl’s puberty book was developed and approved by the government in 2012, with subsequent uptake by the Ministry of Education, UNICEF, and several NGOs. Notably, the government has since requested that the book be translated into twelve additional local languages. Over 400,000 girl’s books have been distributed to date, many of which were used in the Tigray and Afar regions of the country through a partnership program run by the NGO Dignity Period, Mekelle University, and the Miriam Seba menstrual pad factory. Together, the organizations implemented menstrual health and hygiene education programs in schools in the 2 regions that included traveling educators and the provision of books and packages of menstrual products to students. The development of a boy’s puberty book is currently underway after data collection was interrupted due to the COVID-19 pandemic.

### United States Girls’ Puberty Book

After identifying a need among low-income girls for a more meaningful and practical puberty book, the approach was modified to the U.S. context. This included an adaptation effort with girls in Baltimore and formal data collection in the largest, most ethnically diverse cities in the United States (New York City, Los Angeles, and Chicago). Given the complexity of the decentralized education system, the decision was made not to seek national government approval. Following American youth literary trends, the illustrations used were in the style of graphic novels. Due to the COVID-19 pandemic, some book content testing was shifted to videoconferencing. New distribution strategies were utilized for the U.S. context, including the development of complementary animated puberty videos^33^ and partnerships with libraries. Uptake thus far has indicated robust parent or caregiver interest in directly ordering the books to provide to their daughters to read on their own and NGO or community-based organization incorporation of the book into puberty trainings.

### Sierra Leone Girl’s and Boy’s Puberty Books

Two puberty books were published in Sierra Leone in collaboration with CODE, a Canadian NGO, working in partnership with the local government to strengthen teacher training on sexual and reproductive health topics. The COVID-19 pandemic resulted in CODE’s Sierra Leone team leading on engaging the key stakeholders, which included choosing to host a single group meeting rather than conducting individual meetings. One modification made from this local consultation was to also collect peer pressure stories from girls, instead of just boys, for the future puberty book. A virtual training was developed for the local data collection team, including the prerecording of 18 interactive videos due to internet connectivity issues. Real-time technical support and feedback were provided to the local team on WhatsApp. This modified approach provided valuable learning on strategies for remote capacity building. An effort is now underway to evaluate the usefulness of a complementary teacher training guide that was shared along with the books to 260 schools across the country. Primary school teachers received training on puberty and the training guide from CODE, followed by encouragement to incorporate the books into their classrooms or club activities, with brief activities followed by private reading time for the students separated by gender into 2 sides of the room. Formative findings from this simple intervention will be shared in a forthcoming publication.

## LESSONS LEARNED

The process of developing and seeking to scale the puberty books across so many different countries over the last decade has provided valuable insights into what works and does not work for the book development process and distribution strategies.

### Adaptation to the Local Context

It is critically important to adapt each new book to a country context, including local partnerships, inputs from key stakeholders, the capturing of young people’s stories, and the hiring of local illustrators, translators, and publishers. Over the years, numerous requests have been made to “translate” the book for a new country or context (e.g., humanitarian settings). Typically, the approach has been to decline this invitation, given the importance of young people recognizing themselves within the books and of adults in their lives feeling that the books truly capture the country’s growing-up context. Articulating that local partners illustrated and published the book also solidifies a sense of ownership at the country level. This is demonstrated by the numerous additional copies that are ordered and local government approval of the books in almost every country.

### Youth-Driven Research and Peer-to-Peer Learning

The use of participatory methods with girls and boys who are older, aged 16–19 years, is very effective for eliciting young people’s lived experiences. This serves to empower the youth involved as the knowers of their own lives and ensures the content’s authenticity. Older youth can also reflect on their own pubertal experiences and provide wise insights and information for younger children who have yet to reach puberty. In nearly all contexts, the adolescent-written content (e.g., menstrual stories, peer pressure stories) is often the most popular book section among youth readers, who explicitly indicate their appreciation of advice and insights from “real” boys and girls like them.

In nearly all contexts, the adolescent-written content (e.g., menstrual stories, peer pressure stories) is often the most popular book section among youth readers.

### The Value of Stakeholder Buy-in

The importance of local buy-in begins in the first step of the book development process. Contiguous dialogue with key stakeholders throughout the duration of the approach, including gathering their inputs on key topics to include, data collection sites, and content feedback, amplifies the reach of the book upon completion. This has also included carefully field-testing the final written and illustrated content with the key stakeholders. Such engagement has shown to be an effective way for the books to gain government approval in each country and for the scaling of the books. For example, UN agencies and NGOs consulted throughout the process often order large quantities of the final published book(s). Negotiations with stakeholders (e.g., parents/caregivers, curriculum review boards) have illustratively included, for example, using the words “secret areas” in 1 country while another country preferred using the word “vagina.”

### Respect for Sociocultural Norms and Finding a “Middle Ground”

Providing content that meets a “middle ground” socially and culturally within a country has proved effective. Many countries have numerous ethnic groups (e.g., 120 in Tanzania; 80 in Ethiopia). Such diversity means a range of puberty-related traditions, differing clothing and hairstyles, and varying activities for youth in and out of school. This can prove challenging when attempting to develop a nationally accepted and appealing book. Early advice from Tanzanian colleagues, the country where the first book was developed, was to aim for a “middle ground” and recognize that incorporating a true diversity of youth experiences or styles in each country was impossible. That advice, which also includes publishing the first book in the predominant local language and English (when preferred by the government), has proven to be successful. Countries have then adapted as needed, such as Ethiopia, where there are now books in 12 languages, some with modified illustrations.

### Local Ownership: Challenges and Strengths

The intentional decision to not establish a local “office” in any of the countries where the books are developed or a salaried “champion” in each country following the initial government approval phase can create some challenges. Despite documented learning on the acceptability of this content,^21^ the high frequency of staff turnover at NGOs, UN agencies, and government departments coupled with shifts in funding and donors can impact local awareness of the books. This hinders repeated orders for girls and boys coming of age. The assumption was that designing locally appropriate books approved by the government would be sufficient for sustaining ordering; however, that has not always proven to be the case. Despite such challenges, interesting models have emerged in several contexts, such as the previously mentioned Dignity Period in partnership with Mekelle University and the Mariam Seba Factory in Ethiopia. This local partnership sought out the books to distribute with their menstrual management project in the Tigray and Afar regions, with over 400,000 books distributed. Another example is from Sierra Leone through the partnership of CODE, The Association of Language and Literacy Educators, and the government. In this context, the 2 puberty books were distributed to schools across the country with a complementary teacher training guide to support use of the books within the classroom or school clubs.

### Modified Model for Pandemic Use

Lastly, the COVID-19 pandemic provided an opportunity to adapt the traditional model for book development that involved a 2-person in-country team (1 international and 1 local) to work from a distance, as described in the Country Snapshots and Table 1. Key learning from the modified model for use during the pandemic included the effectiveness of a rapidly produced online training of a local team to conduct the participatory data collection despite significant connectivity issues (e.g., 16 prerecorded videos were produced), such as in Sierra Leone. In addition, an essential aspect of the pandemic-adapted approach was having a dedicated local partner—in this case, CODE—to manage the in-country processes (e.g., hiring of the illustrator and publishing company). Although some challenges existed, this example nevertheless highlighted the viability of using a remote technical assistance approach in the future for book development in new countries. In the U.S. context, although the team was able to shift to individual or small group videoconferencing for the book content testing, it limited the number of girls and adults involved in the review process as compared to larger in-person groups used in other country sites.

### Limitations

The research has primarily focused on the puberty experiences of girls and boys. A need remains for improved evidence and documentation on the pubertal development experiences of a broader expanse of gender identities. In addition, the book development model, as currently designed, does not address the needs of youth who are illiterate, although recommendations to various country stakeholders have illustratively included peer partnerships that will enable reading of the book by an older or more literate youth to another who may be illiterate. Lastly, evaluation of the impact of the book when young people read it on their own—the intended aim of the books given the benefit of private learning about the confusing changes of puberty—would serve to benefit decisions around investing in puberty books, any needed improvements in their current content, and additional models for book delivery to young people that respects their frequent discomfort with this topic.

A need remains for improved evidence and documentation on the pubertal development experiences of a broader expanse of gender identities.

## CONCLUSION

The overall learning and importance of the puberty book model that we describe highlights the valuable potential role of puberty books to reach more youth around the world. The rationale for using such an approach relates to: (1) the absence of educational systems that currently are not capacitated to deliver such content or that may deliver content but too late; (2) the importance of keeping youth at the center of developing the content; and (3) the critical value of local partnerships for developing meaningful and culturally acceptable and appropriate books. Finally, much more work is needed to expand the reach of puberty education globally, ranging from developing books in more countries; making sure parents, teachers, and others understand the value of puberty education and feel able to convey it; ensuring content is targeting early adolescents so that they are prepared for the coming changes and feel confident enough to ask for support; and most importantly, making sure youth all over the world have their own puberty book.

## Notes

**Peer Reviewed**

**First published online:** May 12, 2023.
